# Cri du chat syndrome patients have DNA methylation changes in genes linked to symptoms of the disease

**DOI:** 10.1186/s13148-022-01350-3

**Published:** 2022-10-14

**Authors:** Petter Holland, Mari Wildhagen, Mette Istre, Olaug Marie Reiakvam, John Arne Dahl, Arne Søraas

**Affiliations:** Department of Microbiology, Oslo University Hospital, Oslo, Norway

**Keywords:** Cri du chat, 5p minus, Monosomy 5p, Syndrome, DNA methylation, Polycomb, ezh2, h3k4me2, h3k4me27, Disease, Congenital

## Abstract

**Background:**

Cri du chat (also called 5p deletion, or monosomy 5p) syndrome is a genetic disease caused by deletions of various lengths in the short (p) arm of chromosome 5. Genetic analysis and phenotyping have been used to suggest dose-sensitive genes in this region that may cause symptoms when a gene copy is lost, but the heterogeneity of symptoms for patients with similar deletions complicates the picture. The epigenetics of the syndrome has only recently been looked at with DNA methylation measurements of blood from a single patient, suggesting epigenetic changes in these patients. Here, we conduct the deepest epigenetic analysis of the syndrome to date with DNA methylation analysis of eight Cri du chat patients with sibling- and age-matched controls.

**Results:**

The genome-wide patterns of DNA methylation in the blood of Cri du chat patients reveal distinct changes compared to controls. In the p-arm of chromosome 5 where patients are hemizygous, we find stronger changes in methylation of CpG sites than what is seen in the rest of the genome, but this effect is less pronounced in gene regulatory sequences. Gene set enrichment analysis using patient DNA methylation changes in gene promoters revealed enrichment of genes controlling embryonic development and genes linked to symptoms which are among the most common symptoms of Cri du chat syndrome: developmental delay and microcephaly. Importantly, this relative enrichment is not driven by changes in the methylation of genes on chromosome 5. CpG sites linked to these symptoms where Cri du chat patients have strong DNA methylation changes are enriched for binding of the polycomb EZH2 complex, H3K27me3, and H3K4me2, indicating changes to bivalent promoters, known to be central to embryonic developmental processes.

**Conclusions:**

Finding DNA methylation changes in the blood of Cri du chat patients linked to the most common symptoms of the syndrome is suggestive of epigenetic changes early in embryonic development that may be contributing to the development of symptoms. However, with the present data we cannot conclude about the sequence of events between DNA methylation changes and other cellular functions—the observed differences could be directly driving epigenetic changes, a result of other epigenetic changes, or they could be a reflection of other gene regulatory changes such as changed gene expression levels. We do not know which gene(s) on the p-arm of chromosome 5 that causes epigenetic changes when hemizygous, but an important contribution from this work is making the pool of possible causative genes smaller.

**Supplementary Information:**

The online version contains supplementary material available at 10.1186/s13148-022-01350-3.

## Background

Cri du chat syndrome was first recognized by the characteristic high-pitched cry of afflicted babies during their first years of life. The genetic cause is a hemizygous deletion of variable length on the short (p) arm of chromosome 5, first described in 1963 [1]. The incidence is between 1 in 15,000 and 50,000 live births, with a higher prevalence for females (66%) than males, but the reason for this is unclear [2–4]. A recent deep phenotyping study by Nevado et al. [5] defined the most frequent features, symptoms, and comorbidities of the disease by creating a 0–100 score that integrates information about 432 patients from multiple publications. They arrived at the developmental delay (scored 95) and hypotonia (scored 99) as being the two symptoms with the highest scores. Also notable is the typical cry/acute voice (scored 88, 6th most frequent) and microcephaly (scored 65, 14th most frequent). The severity of symptoms appears correlated to the length of the 5p deletion, but the variability between individuals with similar deletions is surprisingly large. A recent study found 39% of Cri du chat patients have additional genetic rearrangements, but they did not find a strong link between having additional genomic changes and distinct symptoms [5].

Large efforts have been put into the genetic mapping of Cri du chat patient’s deletion regions and correlating the deleted regions to symptoms, with the hope of finding dose-sensitive gene(s) that may cause one or several symptoms. Several studies agree on a region relatively near the end of the 5p-arm (cyt.band 15.33) as being causative in the development of the characteristic cry. The telomerase gene (TERT) is found in this region and suggested to be dose-sensitive [6] but it remains unknown if and how it contributes to any of the symptoms. Microcephaly, behavioral, and learning difficulties have been suggested to be caused by hemizygosity of regions further from the end of the 5p-arm (cyt.bands 15.2 and 14.3). Again, the genetic causes have not been definitively determined, but a gene that is known to affect neuronal development, CTNND2, is often suggested to contribute [7].

The epigenetics of Cri du chat syndrome is unexplored, except for the DNA methylation mapping of one patient, where blood from a single toddler with the syndrome was assessed [8]. DNA methylation measurements of blood cells to characterize people by so-called episignatures is an approach with a rapidly growing number of applications. Most famous is the determination of biological age [9], demonstrated to be correlated with the risk of a range of diseases [10]. More recently, DNA methylation episignatures of the blood have also been used to classify and stratify patients with genetic syndromes [11, 12]. In this manuscript, we report a deep analysis of DNA methylation in a cohort of eight Norwegian Cri du chat patients. Because DNA methylation is well known to change with aging, we collected blood from closely aged siblings as controls wherever possible and age-matched non-related controls otherwise. We discover a distinct pattern of DNA methylation changes in gene regulatory sequences with enrichment for the disease categories of the most common Cri du chat symptoms. This suggests that Cri du chat symptoms may be driven by epigenetic mis-regulation during development.

## Results

**Fig. 1:**
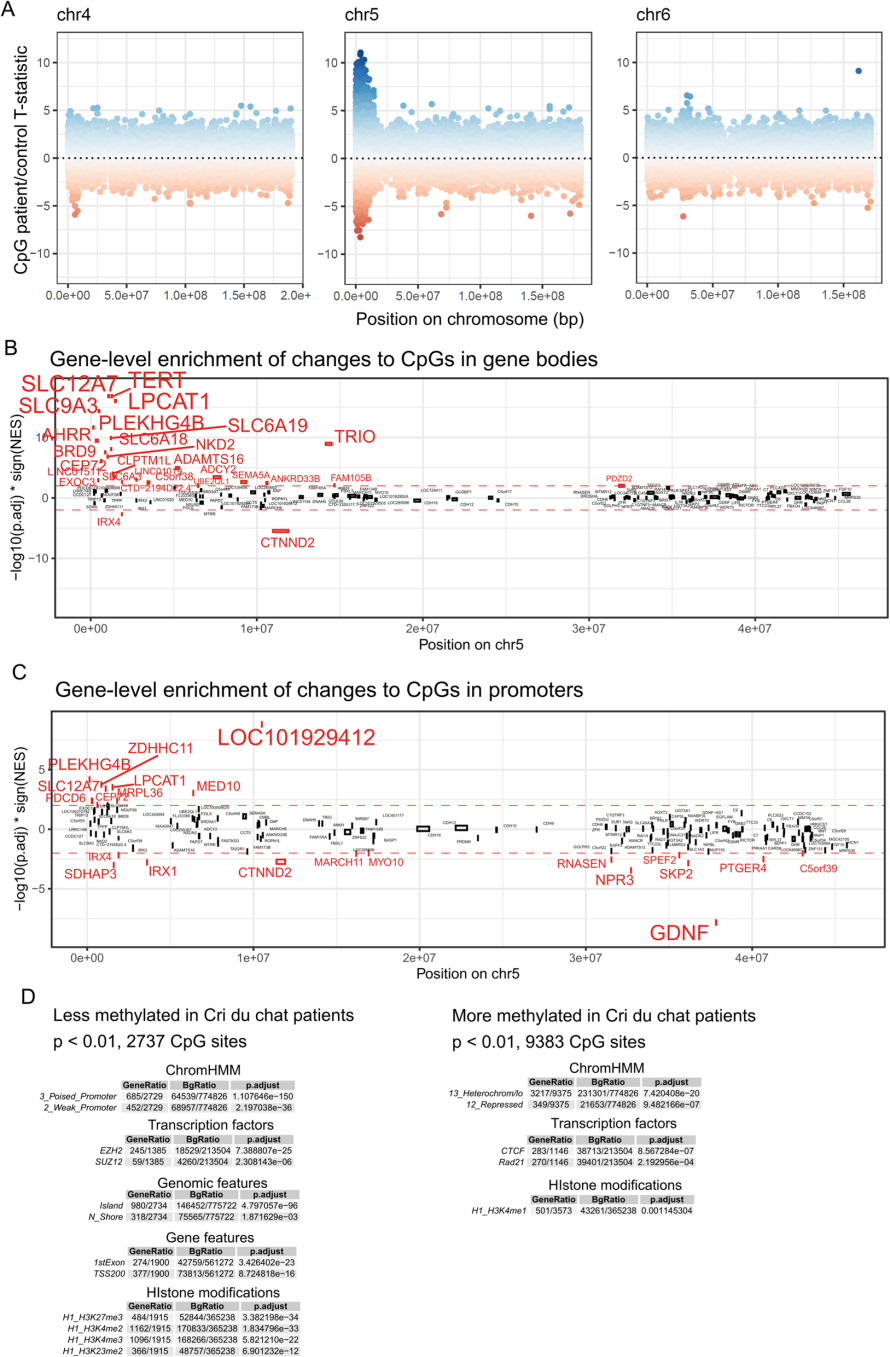
DNA methylation changes and functional enrichment in Cri du chat patients. **A** Moderated T-statistic for each CpG site calculated from the paired patient/controls and mapped to genomic positions. Here shown for chromosomes 4–6. All chromosomes are shown in Additional file 1: Figure S1. **B**, **C** Integration of patient CpG changes on the p-arm of chromosome 5 relative to controls for gene bodies (**B**) or promoter sequences (**C**). The *y*-axis shows the −log10(*p*.adjusted) of the statistical test measuring the likelihood of patient DNA methylation of that given promoter (or gene body) is be differentially methylated. It is multiplied by the sign of the NES value, meaning strongly positive *y*-axis values would be significantly increased methylation. The dotted red lines are at *y* = −2 and 2, representing *p*.adj < 0.01. All gene bodies or promoters with *p*.adj < 0.01 are in red with increasing font size for lower *p* values. **D** Enrichment analysis with several different types of CpG categorization for the CpGs with *p* < 0.01 and separated CpGs with less or more methylation in patients

DNA methylation was measured at 850,000 CpG sites from blood samples of eight Cri du chat patients and matched controls by microarray. After removing CpG probes with low detection *p* values, those on *X* and *Y* chromosomes, and those linked to single-nucleotide polymorphisms, 786,010 CpG sites remained. We applied a statistical test for each CpG site, comparing eight paired patients and controls. The paired T-statistic is shown for all CpGs mapped to their genome-wide location is shown in Additional file 1: Figure S1, with chromosome 5 and two representative comparison chromosomes shown in Fig. 1A. We performed an unbiased characterization of the samples using principal components analysis (PCA) to look for any systematic bias that may be affecting the samples (Additional file 1: Figure S2A). The principal component best separating the samples (PC1) separates samples according to age at sampling, with the older patients and controls to the left and youngest patients and controls to the right. To reduce the possibility of personal identification, we have excluded personal information such as the age at sampling and gender in Additional file 1: Figure S2A, but we show the age difference between the samples at the time of sample measurement and sibling relationships between paired samples in Table 1. The second-best principal component (PC2) does not appear to be integrating any of the other covariates of the samples such as gender, sample type (dried blood spot or EDTA tubes), array position, or DNA concentration. We conclude that by comparing age-matched patients and controls, we can arrive at a comparison of Cri du chat DNA methylation without any apparent systematic bias.

**Table 1:** Details about the age difference at blood sampling and genetic relationship between patients and controls.

Set	Control	Patient	Age difference at sampling (years)	Siblings	Notes
1	c1	p1	0.14	No	
2	c2-1, c2-2	p2	0.2, 7.6	No, Yes	Age-matched non-sibling and sibling. Results are mean (p2/c2-1, p2/c2-2)
3	c3	p3-1, p3-2	2	Yes, Yes	Two p3 samples from the same individual, but collected by DBS or EDTA blood sampling. Results are mean (p3-1/c3, p3-2/c3)
4	c4	p4	0	No	
5	c5	p5	0.5	Yes	
6	c6	p6	0.002	No	
7	c7	p7	1.4	No	
8	c8	p8	3.7	No	

Blood samples were collected over a span of several years, meaning that the age difference at sampling is not representative of the actual age difference between individuals measured

Although Cri du chat patients generally don’t have immunodeficiencies, if there are slight differences in immune cell populations in the patients, this can bias DNA methylation comparisons between patients and controls. We applied a well-established algorithm [13] to estimate immune cell populations in the blood and found no differences between patient and control samples (Additional file 1: Figure S2B). The hemizygous deletion of TERT also made us curious to measure the rate of biological aging through DNA methylation because single-nucleotide polymorphisms in TERT have recently been linked to changes in biological aging [14]. While Cri du chat patients are shown to have shorter telomeres, we did not detect any change in biological age by the Horvath multi-tissue 2013 clock [9], the more recent “skin-and-blood” clock [15], the GrimAge clock [10], or a “DNAmTL” clock trained on telomere lengths [16] in the patient samples compared to controls (Additional file 1: Figure S2C).

In Fig. 1A, there is an apparent increase in DNA methylation variability along the p-arm of chromosome 5 (the region that is hemizygous in patients) compared to the rest of the genome. We next explored to what degree these changes in DNA methylation affects genes positioned on the p-arm of chromosome 5. By integrating all the CpG sites in a given gene and performing a ranked gene set enrichment analysis, we can obtain a *p* value that represents how likely it is that the contained CpG methylation changes for a given gene are random. The algorithm also gives a Normalized Enrichment Score (NES) which will be negative if there is less methylation and positive if there is more methylation in the patients. In Fig. 1B, we integrate CpG methylation changes in patients for gene bodies and show the *p* value of enrichment (adjusted for multiple testing) multiplied by the sign of the NES to show the strength of methylation changes as well as the direction of change along the chromosome 5p-arm. There is a tendency for increased DNA methylation in gene bodies on the hemizygous chromosome arm in the patient samples (Fig. 1B). This observed increase in gene body DNA methylation on the hemizygous 5p-arm may be a reflection of increased gene expression to compensate for the loss of a gene copy. Interestingly, when doing this analysis for CpG’s in gene regulatory promoter sequences (Fig. 1C), it is clear that this increased methylation is not indiscriminate along the chromosomal arm because promoter CpG’s appear less affected in the patients.

To look for common patterns among CpG sites that are more or less methylated in patients compared to controls, we performed a relative enrichment analysis using several databases with different categorizations of CpG sites. CpG’s with decreased DNA methylation showed strong enrichment for promoter sequences, CpG islands, transcription factors EZH2 and SUZ12, as well as histone modifications H3K4me2, H3K4me3, H3K27me3, and H3K23me2 (Fig. 1D). Sites with increased DNA methylation were generally enriched for being in heterochromatin and somewhat enriched for H3K4me1 binding sites and binding of the transcription factors CTCF and Rad21 (Fig. 1D). Additional enrichment analysis using other categorization databases of CpG’s is included in Additional file 1: Figure S3A–B. We also performed an enrichment analysis excluding CpG sites on the p-arm of chromosome 5 to see to what degree the enrichment effects are driven by changes to the hemizygous region itself. In general, there is stronger enrichment in categories when CpG sites on the p-arm of chromosome 5 are excluded from the enrichment analysis (Additional file 1: Figure S3C–D), indicating that the patient DNA methylation changes on the p-arm of chromosome 5 may be more indiscriminate and/or driven by different processes than the changes in the rest of the genome.

**Fig. 2:**
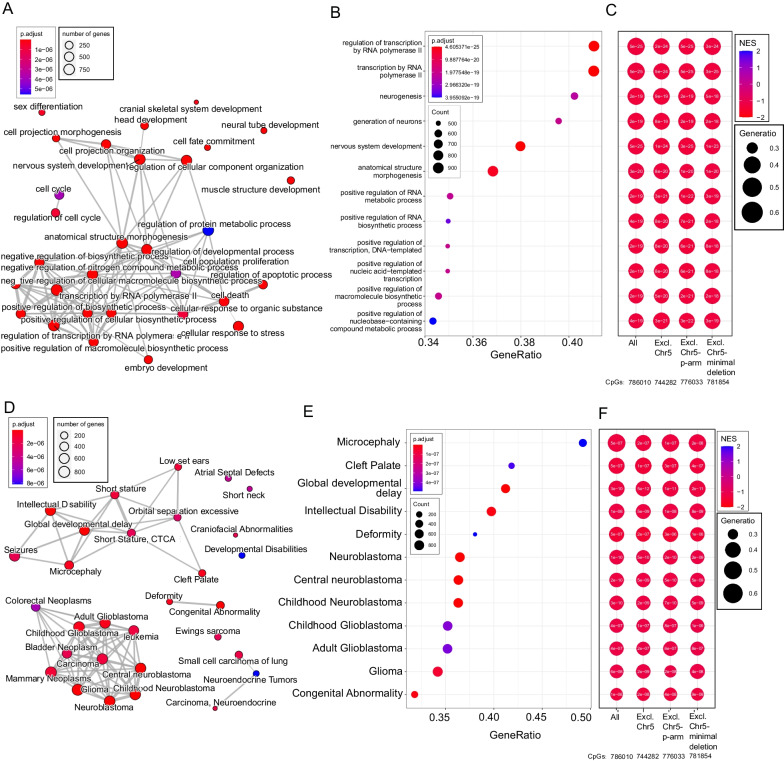
Gene Ontology (GO) and DiseaseGeNET (DGN) enrichment from Cri du chat patient promoter CpG changes. Promoter enrichment -log10(*p*.adjusted)*sign(NES) values that indicate how changed the CpG methylation is in patients compared to controls for a given gene promoter were used as input for gene set enrichment analysis in GO (**A**–**C**) and DGN (**D**–**F**) databases. In (**A**), semantically highly similar groups were combined through the clusterProfiler::simplify function. In (**C**) and (**F**) the promoter enrichment analysis and following GO or DGN enrichment was repeated with reduced set of CpGs, removing different segments of chromosome 5. The color indicates the NES values in (**C**) and (**F**), red indicating less CpG methylation in patients and blue indicating more, and the numbers show the *p*.adj of enrichment

The integrated CpG promoter DNA methylation changes for each gene (−log10(*p*.adjusted) * sign(NES) values) can further be applied to another ranked gene set enrichment analysis of genes where functional categorizations of genes can be tested for enrichment of changes in patients versus controls. By this method, we can obtain an estimate of how likely it is that a category of cellular function is affected by the changes in DNA methylation. Gene Ontology (GO) categorization of genes revealed strong enrichment for a network of cellular processes related to nervous system development (*p*.adj < 5e−25) (Fig. 2A−B) and other organ development and specification processes were also highly enriched (Fig. 2A). Another interesting type of gene categorization is DiseaseGeNET (DGN), an extensive database containing various types of evidence linking genes to human diseases, symptoms and comorbidities. In this database, the strongest enrichment of DNA methylation changes in patients was for Global developmental delay (*p*.adj < 3e−10) and Microcephaly (*p*.adj < 5e−7) (Fig. 2D–E). It was very striking to us that two of the most common symptoms of Cri du chat patients were of the most strongly enriched categories in this analysis.

To test if the relatively large changes we see in DNA methylation of the 5p-arm (Fig. 1A) was the driver of the observed enrichments in Fig. 2, we repeated the gene set enrichment analyses (CpG to gene promoter, then gene to functional category) while excluding different sets of CpG sites on chromosome 5. We find that the exclusion of CpGs in the minimal Cri du chat-deleted region, the whole 5p-arm, or all of chromosome 5 all show a minimal effect on the enrichment results of either Gene Ontology (Fig. 2C) or DGN (Fig. 2F). This demonstrates that the enrichments seen in Fig. 2 are not directly caused by changes in DNA methylation only on chromosome 5, but is caused by DNA methylation changes distributed across the genome. In Fig. 2C, F, the color represents the normalized enrichment score (and numbers FDR-adjusted *p* values), where red represents a negative enrichment score—indicating less methylation in the patients than in controls. The gene set enrichment analysis was also performed with statistical adjustment for the covariates of sex, age, and estimated immune cell populations (Additional file 1: Figure S4A–D). In general, the adjustment for covariates had only small effects on the enrichment results and the most relevant enrichment categories related to neurogenesis, microcephaly, and developmental delay were strongly enriched also when adjusting for covariates. We explored in more detail how the DNA methylation was changing in the patients of some selected categories in Additional file 1: Figure S5A-C. In the categories enriched for having less DNA methylation in the patients, the baseline level of DNA methylation was low and further decreased in the patients (Additional file 1: Figure S5C). The majority of categories showed enrichment because of reduced methylation in patients, but we also demonstrate an example of a category with more methylation in patients in Additional file 1: Figure S5C, and in this case, CpGs have relatively high levels of methylation that is further increased in patients.

**Fig. 3:**
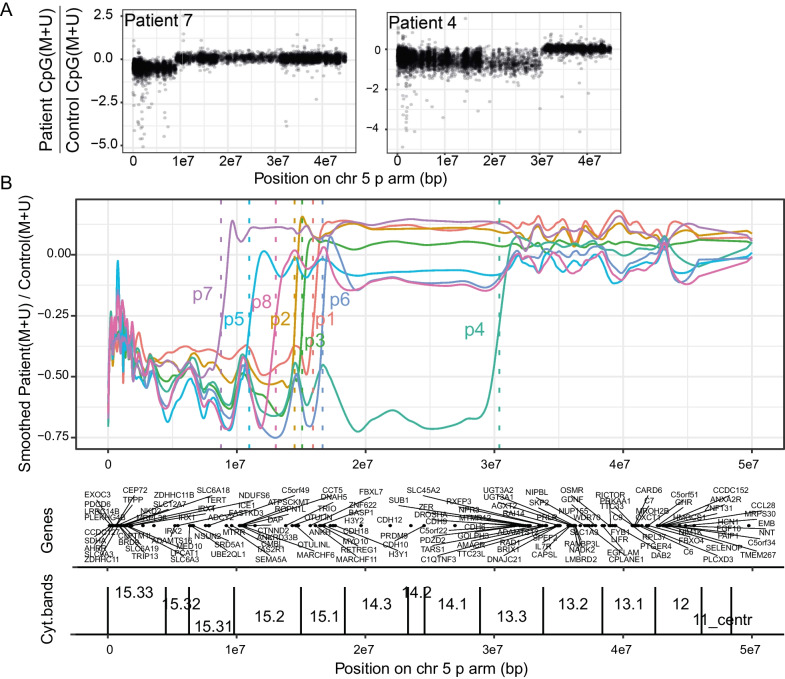
Characterization of the chromosomal deletion from DNA methylation data. **A** All CpG sites on the chromosome 5p-arm for two representative patients with either short (patient 7) or long (patient 4) deletions. The *y*-axis shows the patient sum of methylated and unmethylated signal divided by the mean control methylated and unmethylated sum. **B** Loess smoothing applied to the signal shown in (**A**), defining the estimated deletion point with a dotted vertical line. Gene positions and cytogenic bands are also shown for the indicated part of chromosome 5

Because the DNA microarray gives a measurement of the total methylated and unmethylated signal for CpG probes along the chromosomes, we were curious to see if we could detect where the Cri du chat patients had the hemizygous deletion by summing the methylated and unmethylated signal along the chromosome. We found an apparent decrease in the summed signal at a point along the 5p-arm for each patient when compared to controls (Fig. 3A, Patient 7 representative of short deletion and Patient 4 of long deletion), and by loess smoothing these signals, we could define an approximate deletion point for each patient (dotted vertical lines in Fig. 3B).

**Fig. 4:**
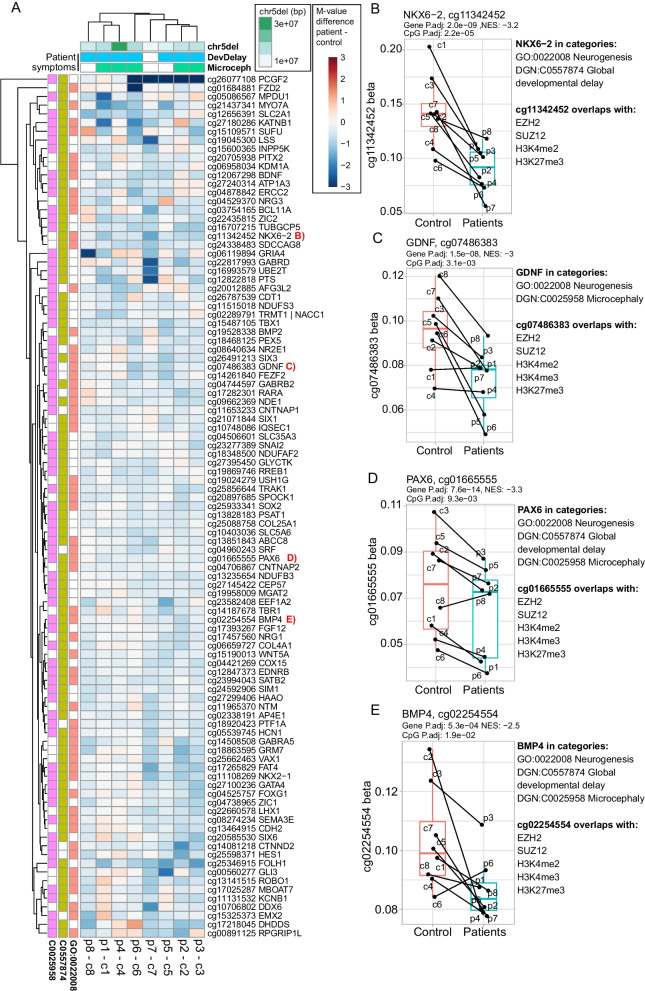
Heatmap of patient–control comparisons for CpGs that are common to minimum 2 of 3 selected categories. **A** On the left relationship to the gene categories is indicated, GO:0022008—Neurogenesis, DGN:C0557874—Global developmental delay. DGN:C0025958—Microcephaly. 373 genes were common to minimum 2 of 3 of the categories and the top 100 of these genes are shown in the heatmap by the CpG that is most strongly changed in the patients/controls. Colors in the heatmap indicate the difference in (log2) *M* values between patient and matched control. On the top of the heatmap is the size of the patient deletion (calculated from Fig. 3A) and relevant symptoms as reported by a patient questionnaire. **B**–**E** Beta values of selected CpGs who are also associated with bivalent marks are demonstrated

To further look for causes of the observed enrichment we focused on three categories that we can link to comorbidities common for Cri du chat syndrome patients—neurogenesis, global developmental delay, and microcephaly. We took the genes in each category that have changes in promoter DNA methylation in the patients (leading edge genes) and created a subset of these genes which are included in minimum 2 of the 3 categories, 373 genes in total. We further extracted the CpG’s that are driving the gene promoter enrichment (leading edge CpGs) and selected the one with the strongest changes in patients (largest absolute t-statistic). In Fig. 4A we show the top CpGs of the top 100 most strongly changed genes from this set of 373 along with the size of the deletion for each patient (calculated from the dotted lines in Fig. 1B) and reported patient symptoms on the top of the heatmap.

In the analysis shown in Fig. 1D we found that CpG’s with decreased methylation in the patients were enriched for several marks of bivalent promoters; EZH2, SUZ12, H3K27me3, H3K4me2, and H3K4me3. Based on the observation that the selected gene categories were also generally less methylated (Additional file 1: Figure S5C), we were curious to see how many of the 100 highlighted CpG’s in Fig. 4A are bivalent promoters. We found that 24 of 100 CpG’s shown in Fig. 4A contained minimum 4 out of 5 of the bivalent marks (EZH2, SUZ12, H3K27me3, H3K4me2, H3K4me3) while 11 out of 100 contained 5 out of 5. In Fig. 4B we show the beta values of the individual samples in our analysis for some selected CpG’s that are in bivalent promoter regions. EZH2 mutations are known to be able to cause a syndrome called Weaver’s syndrome. In a recent publication, DNA methylation was mapped for a set of Weaver’s syndrome patients [17] and a list of 229 CpG’s were defined as being highly significant between patients and controls, with a majority showing reduced methylation in the patients. When comparing the DNA methylation signal in our Cri du chat patients compared to controls to the Weaver’s syndrome patients signal relative to controls, we find a striking negative correlation in the CpGs that are significantly different for Weaver’s syndrome patients (*R* = −0.54, Additional file 1: Figure S6), further suggestive of a functional link between Cri du chat syndrome and bivalent promoters.

## Discussion

The most striking finding from our analysis is the enrichment of changes in DNA methylation in gene promoters that are linked to several of the best-known symptoms afflicting Cri du chat syndrome patients. While it is tempting to infer a causal link between observed DNA methylation changes and patient symptoms, this inference is premature with the current data and the patterns of changed DNA methylation should presently be treated as a Cri du chat DNA methylation signature. After excluding that the symptom-category enrichment is driven directly by changes in methylation of genes on chromosome 5, we speculate that the loss of one or several dose-sensitive genes on chromosome 5 causes changes to developmental programs during embryo development, possibly contributing to the development of patient symptoms. The epigenetic signature that we see in the blood DNA methylation data could be driving these changes by affecting development, but the changes could also be secondary, resulting from other changes that affect development. In either case, the epigenetics of Cri du chat syndrome should be studied further to hopefully get closer to causative mechanisms.

The enrichment of components of the polycomb repressive complex, EZH2 and SUZ12, is interesting in this context. EZH2 is a histone methyltransferase that methylates H3K27me, contributing to gene repression [18]. EZH2 mutations in Weaver’s syndrome cause characteristic DNA methylation changes [17]. The fact that we see a negative correlation between Cri du chat patient’s methylation and Weaver’s syndrome methylation suggests that EZH2 activity may somehow be changed in the Cri du chat patients.

If we assume that the loss of one copy of the 5p-arm causes epigenetic changes early in embryo development, a good place to start to find which genes are causative in driving the Cri du chat symptoms is to look for genes linked to epigenetic functions. It has been previously suggested that MTRR (in the 15.31 region of 5p), a gene linked to folate and methionine metabolism that contributes to the supply of methyl groups for DNA methylation, may have a role in the pathogenesis of Cri du chat syndrome [8]. Loss of one copy of MTRR has in fact been shown to affect DNA methylation in mice, with transgenerational effects [19]. Another candidate epigenetic regulator that is mostly unexplored in the context of Cri du chat is BRD9 (in the 15.33 region of 5p). BRD9 acts partly together with CTCF, playing a role in the regulation of the 3D organization of the genome. BRD9 has in fact been shown to regulate EZH2 expression by binding together with CTCF in an EZH2 enhancer [20, 21], but we do not know if EZH2 expression changes contribute to Cri du chat syndrome pathogenesis. It is notable that we find enrichment of CTCF binding sites in the CpGs with increased methylation in patients (Fig. 1D), suggesting the possible functional sequence BRD9→CTCF→EZH2. BRD9 also affects genome stability [22] and thus loss of BRD9 could also contribute to the observed increased rate of genomic rearrangements in Cri du chat patients.

A limitation of our study is the fact that we are analyzing data from blood cells, which is not a tissue that is functionally changed in Cri du chat patients. When we find patterns of change in blood, we think it is likely that similar DNA methylation changes exist also in other functionally affected patient tissues, but this remains to be demonstrated. Our study design also does not allow us to conclude anything about the sequence of events leading to and resulting from the observed DNA methylation changes. We hope future studies of Cri du chat epigenetics can look for similar effects in an independent patient population, with a different measurement technology like bisulfite sequencing, and explore other experimental strategies to shed light on mechanistic details.

## Conclusions

By performing the deepest epigenetic study of Cri du chat to date, with age- and sibling-matched paired patient controls, we detect DNA methylation changes on gene promoters linked to symptoms affecting Cri du chat patients. However, we cannot conclude what is causing the observed epigenetic changes and whether they causally contribute to adult Cri du chat patient symptoms. The most important contribution of our study to the knowledge about Cri du chat syndrome is to suggest the involvement of epigenetics, giving direction for future mechanistic studies to understand this understudied syndrome.

## Methods

### Patient recruitment, blood sampling, and DNA methylation arrays

Patients with Cri du Chat and their siblings were recruited through a social media group for Cri du Chat relatives in Norway. Newborn non-sibling controls were recruited at the maternity ward at Oslo University Hospital, Rikshospitalet. Dried Blood Spot (DBS) samples were collected from participants using Whatman (LIPIDX) collection cards. In some cases, extracted DNA available in other biobanks at Oslo University Hospital enabled DNA collection without the need for participant sampling. DNA methylation was measured on Illumina EPIC arrays by Life and Brain GmbH, Bonn, Germany.

### DNA methylation raw data processing

The array fluorescence measurements were imported into R with the minfi library [23]. Detection *p* values were calculated by minfi::detectionP and CpGs with *p* < 0.01 for all samples were included for further analysis. minfi::qcReport was inspected and showed no outlying samples (see also the PCA plot in Additional file 1: Figure S2A) or systematic differences between controls and patients. Arrays were then normalized by minfi::preprocessNoob. Probes found to be off-target or polymorphic by McCartney et al. [24] were excluded and then DMRcate::rmSNPandCH was used to remove further SNPs and sex chromosome probes. After these steps, 786,010 CpGs remained for further analysis. Tables of total signal sums were exported by sum(minfi::getMeth,minfi::getUnmeth), tables of beta values by minfi::getBeta and tables of M values by minfi::getM.

### Finding the patient chromosome deletion from the sum of array signals

For each patient sample, calculate log2 of the sum(methylated,unmethylated) signal divided by the mean of all controls sum(methylated,unmethylated) for each CpG. To define the point of deletion, smooth a loess line to this signal with span = 0.05. The deletion point was determined to be the position farthest out on the p-arm of chromosome 5 (lowest *x*-axis value) that is larger than 5e6 (due to noise near the end of the chromosome) and has a higher smoothed signal than −0.25.

### Estimating blood cell populations

Proportions of different blood cells were estimated through the FlowSorted.Blood.EPIC [13] R package using the estimateCellCounts2 function with default parameters.

### DNAm biological age estimation

For the biological clocks, the following reference sets of CpGs were used to calculate the biological age of the samples: Horvath_2013 [9], Skinblood [15], DNAmTL [16], and GrimAge [10]. The GrimAge CpG sites are not publically known, but we obtained from the authors a stand-alone python implementation of the clock that we executed locally to be GDPR compliant.

### CpG site T-statistic calculation

Each CpG site was compared between patients and controls by using the mCSEA::rankProbes [25] function with a paired analysis. mCSEA uses the well-established limma + eBayes pipeline to compute moderated t-statistics. Adjustment for confounders was performed by including them in the execution of mCSEA::rankProbes.

### Promoter or gene body enrichment analysis

All CpGs were ranked by their T-statistic and applied to gene set enrichment analysis (GSEA) through the mCSEA R package [25]. Databases linking CpGs to gene bodies or gene promoters for EPIC arrays are contained in the mCSEA package.

### Functional enrichment categories

Several databases were assembled to search for significant enrichment of different types of functional categorizations of CpGs. chromHMM and transcription factor enrichment databases for EPIC arrays were obtained from Ref. [26]. Transcription factor analysis was limited to data from H1 embryonic stem cells. Position of a CpG relative to island, shores, or position within a gene was contained in the Illumina array annotation file. Histone modification data for H1 embryonic stem cells was obtained from the ENCODE database. Additional gene-level databases are the C1, C2, and C3 human databases contained in msigdbr [27]. Enrichment analysis was performed by the clusterProfiler::enricher [28] with parameters minGSSize = 4, maxGSSize = Inf, and the universe of CpGs that enrichment is performed relative set to the 786,010 CpGs included in the patient–control comparison.

### Gene ontology and DiseaseGeNET analysis of promoter enrichment

From the enrichment scores of patient CpG changes in promoters, a Normalized Enrichment Score (NES) is given, ranking all promoters according to how large the changes are in each direction, either more methylation in the patients (positive NES) or less (negative NES). The sign of the ranked promoter NES scores is multiplied by the -log10(*p*.adjusted) of that given promoters enrichment to create a score that includes the direction of change as well as significance of enrichment per promoter which is further applied to a gene-set enrichment analysis by the clusterProfiler R package [28] with the gseGO and gseDGN functions. In the control analysis when different parts of chromosome 5 were removed from this enrichment analysis, the rankProbes function was rerun with the reduced set of CpGs, and promoter sign(NES)*−log10(*p*.adj) was calculated from this set of probes and then this ranked set applied to gseGO and gseDGN.

## Supplementary Information

**Additional file 1**. Supplementary Figures 1–6.

## Data Availability

Anonymized DNA methylation raw data can be found at EMBL ArrayExpress accession E-MTAB-12302.

## Abbreviations

BP: Base pairs
*P*.adj: *P* Value adjusted for multiple testing
ES: Enrichment score
NES: Normalized enrichment score
TSS: Transcription start site
CpG: CG-dinucleotide where the C is commonly methylated
EZH2: Enhancer of zeste homolog 2
CDC: Cri du chat
GDNF: Glial cell line-derived neurotrophic factor
BRD9: Bromodomain-containing protein 9
CTCF: CCCTC-binding factor
