# Supplementary material for: Cri du chat syndrome patients have DNA methylation changes in genes linked to symptoms of the disease
Source: Clin Epigenetics. 2022 Oct 14;14:128. doi: 10.1186/s13148-022-01350-3 (PMC9563797; doi:10.1186/s13148-022-01350-3)
Supplement: Supplementary file 1 — Additional file 1. Supplementary Figures 1–6. [file 13148_2022_1350_MOESM1_ESM.docx]

**Supplementary Figure 1:** T-statistic for a paried patient/control comparison, all CpGs genome wide.

**Supplementary Figure 2:** a) Principle component analysis of beta values for all samples analyzed. The four rows for each sample is 1: ArrayNumber_ArrayPosition. 2: Dried blood spot (DBS) or EDTA whole blood. 3: Patient or control identifier. 4: DNA concentration. Additional details on the samples are given in Table 1. b) Immune cell relative composition, calculated from DNA methylation data by the Houseman method. c) DNA methylation biological clocks measured for four different clocks.

**Supplementary Figure 3:** a,b) Enrichment analysis for p<0.01 of individual CpGs that are less methylated (a) or more methylated (b) in patients. Same sets of CpGs as in Figure 1D, but for additional enrichment databases. c,d) Enrichment analysis for p<0.01 of individual CpGs that are less methylated (c) or more methylated (d) in patients, but excluding all CpGs on the p-arm of chromosome 5.

**Supplementary Figure 4:** Enrichment analysis of Cri du chat patient promoters, adjusted for the covariates of sex, age and immune cell populations. a) Gene ontology and b) DiseaseGeNet top categories from the analysis which is not adjusted for covariates is shown, together with NES (color), generatio (size) and FDR-adjusted p.value (text inside circle). c,d) Venndiagrams for the top 100 enriched categories from the non-adjusted gene ontology (c) and diseasegenet (d) showing if they are also found in the top 100 after adjusting for covariates.

**Supplementary Figure 5:** a) GSEA plots of two example gene promoter sets which are strongly enriched in patients. b) GSEA plot of an example enriched DGN category, gene promoters are ranked by sign(NES)*-log10(p.adj) c) Highlighted categories from Figure 2, showing the category names and the beta as well as M values for the leading edge CpGs (those that contribute to the significant enrichment) in these categories. C3501844 (Familial Nonmedullary Thyroid Cancer) is included to show the relation between methylation in patients and controls in a category that has increased methylation in the patients.

**Supplementary Figure 6:** From Supplementary Table S4 in Choufani et al (American Journal of Human Genetics, 2020), the most strongly significantly changed CpGs in Weaver syndrome patients and their beta value differences in patients-control were obtained. The beta value differences are plotted together with the T-statistic of the same CpG sites from the Cri du chat patient compared to control.
